# Head and Neck Squamous Cell Carcinoma: Insights from Dual-Energy Computed Tomography (DECT)

**DOI:** 10.3390/tomography10110131

**Published:** 2024-11-11

**Authors:** Eleonora Bicci, Antonio Di Finizio, Leonardo Calamandrei, Francesca Treballi, Francesco Mungai, Stefania Tamburrini, Giacomo Sica, Cosimo Nardi, Luigi Bonasera, Vittorio Miele

**Affiliations:** 1 Department of Radiology, Careggi University Hospital, 50134 Florence, Italy; f.mungai@gmail.com (F.M.); cosimo.nardi@unifi.it (C.N.); bonaseral@aou-careggi.toscana.it (L.B.); vmiele@sirm.org (V.M.); 2 Department of Radiology, Careggi University Hospital, University of Florence, 50134 Florence, Italy; antonio.difinizio@unifi.it (A.D.F.); leonardo.calamandrei@unifi.it (L.C.); francescatreballi@gmail.com (F.T.); 3 Department of Radiology, Ospedale del Mare, ASL NA1 Centro, 80147 Naples, Italy; tamburrinistefania@gmail.com; 4 Department of Radiology, Monaldi Hospital, 80131 Naples, Italy; sicagf@libero.it; 5 Department of Experimental and Clinical Biomedical Sciences, University of Florence, 50134 Florence, Italy

**Keywords:** computed tomography, DECT, dual-energy CT, head and neck, oncology

## Abstract

Head and neck cancer represents the seventh most common neoplasm worldwide, with squamous cell carcinoma being the most represented histologic variant. The rising incidence of the neoplastic pathology of this district, coupled with the drastic changes in its epidemiology over the past decades, have posed significant challenges to physicians worldwide in terms of diagnosis, prognosis, and treatment. In order to meet these challenges, a considerable amount of effort has been spent by the authors of the recent literature to explore new technologies and their possible employment for the better diagnostic and prognostic definition of head and neck squamous cell carcinoma (HNSCC). Among these technologies, a growing interest has been gathering around the possible applications of dual-energy computed tomography (DECT) in head and neck pathology. Dual-energy computed tomography (DECT) utilizes two distinct X-ray energy spectra to obtain two datasets in a single scan, allowing for material differentiation based on unique attenuation profiles. DECT offers key benefits such as enhanced contrast resolution, reduced beam-hardening artifacts, and precise iodine quantification through monochromatic reconstructions. It also creates material decomposition images, like iodine maps, aiding in tumor characterization and therapy assessment. This paper aims to summarize recent findings on the use of DECT in HNSCC, providing a comprehensive overview to aid further research and exploration in the field.

## 1. Introduction

HNSCC is the sixth most common malignancy worldwide, accounting for approximately 6% of all cancer cases and responsible for an estimated 1–2% of all cancer deaths [1,2,3,4]. The pathological mechanism of HNSCC involves a complex interplay of genetic mutations, environmental factors, and viral infections [1,2,3,4]. Among these, smoking and alcohol consumption are recognized as the principal risk factors [1,2,3,4]. Another important risk factor for HNSCC is infection with the Human Papillomavirus (HPV) [5]. HPV-related HNSCC is more common in the oropharynx and has a distinct clinical and molecular profile compared to those caused by smoking and alcohol [4,5].

The diagnosis of HNSCC typically involves a combination of clinical examination, imaging studies, and biopsy [2,4]. Imaging modalities such as CT, MRI, and PET/CT play a crucial role in the staging and management of HNSCC [1,3,4,5]. Treatment options vary based on the stage and location of the tumor and may include surgery, radiation therapy, chemotherapy and targeted therapies [2,3,4,5]. The prognosis for HNSCC patients depends on several factors, including the tumor’s stage, location, and HPV status [1,2,3,4,5]. 

Since the launch of clinical dual-energy CT (DECT) scanners, there has been a growing interest in research about the benefits of using DECT for evaluating the head and neck pathology, particularly for head and neck squamous cell carcinoma (HNSCC) [6,7]. The aim of this paper is to summarize recent findings related to the application of DECT in HNSCC. This synthesis is intended to serve as a valuable resource for those seeking a comprehensive overview of the subject, with the goal of providing useful insights for further exploration through new studies. 

Dual-energy computed tomography (DECT) involves the use of two distinct X-ray energy spectra, enabling the acquisition of two datasets during a single scan [8,9]. This dual-energy approach allows for the differentiation of materials based on their unique attenuation profiles at different energy levels, providing additional diagnostic information compared to single-energy CT [10,11,12,13]. There are several techniques for implementing DECT, including dual-source CT, rapid kVp switching, dual-layer detector CT, and dual-spin CT. Each technique has its advantages and limitations, and the choice of technique depends on the specific clinical application. One of the key advantages of DECT is the ability to perform monochromatic reconstructions [14,15]. These are images reconstructed at a single energy level, which can be selected from a wide range of energies. Monochromatic images can improve contrast resolution, reduce beam-hardening artifacts, and provide a more accurate quantification of iodine concentration in contrast-enhanced studies [16,17]. DECT also allows for the creation of material decomposition images or maps [12,18,19], which can differentiate and quantify specific materials within the body. For example, iodine maps can provide a quantitative measure of iodine uptake in tissues, which can be useful in tumor characterization and in assessing the response to therapy [20,21,22,23,24]. Several DECT-derived parameters are used in the analysis of radiological images [8,11,18,25,26]. These include the normalized iodine concentration (NIC), which provides a measure of iodine uptake normalized for the blood pool; the slope of the spectral Hounsfield unit curve (λHU), which reflects the energy dependence of attenuation and can be used to differentiate materials; and the effective atomic number (Zeff), which provides information about the atomic composition of tissues and can further help in material differentiation.

## 2. DECT in Cancers of the Nasopharynx

Nasopharyngeal carcinoma (NPC) is a rare cancer in the group of head and neck neoplasms, known to have a particular epidemiology, being endemic in North Africa and Southeast Asia, with a low incidence in Europe [27,28,29]. Squamous cell carcinoma (SCC) is the most frequent histotype [30,31,32]. Nasopharyngeal squamous cell carcinoma (NPSCC) is classified by the WHO into three subtypes: nonkeratinizing squamous cell carcinoma (differentiated and undifferentiated subtype), keratinizing squamous cell carcinoma, and basaloid squamous cell carcinoma, with Epstein–Barr Virus (EBV) and Human Papillomavirus (HPV) being known to be implicated in pathogenesis [33,34]. The tumor generally originates in the fossa of Rosenmüller, at the level of the posterolateral wall of the pharynx, which is why unilateral hearing loss due to the obstruction of the Eustachian tube ostium is often the onset symptom of the neoplasm [27,29,33,34,35,36]. Prognosis depends on the extent of the tumor and lymph node involvement at staging, histology, and biomarkers, such as the presence of the virus, which is usually associated with a better prognosis [32,35]. Treatment involves the use of radiotherapy combined or not with chemotherapy, while surgery has a limited role [35,37,38,39,40,41,42]. MRI is generally the imaging technique of choice for staging, given the better definition of the morphologic parameters of the neoplasm due to its high-contrast resolution, but it is often accompanied by complementary imaging techniques, mostly CT and PET [43,44,45,46,47]. Not many studies that have evaluated the potential of spectral imaging with DECT have applied to the study of nasopharyngeal cancers, but, recently, some works have brought interesting results to light. 

In a retrospective study comprising 80 patients, Shen et al. attempted to evaluate whether DECT could help in the non-invasive differential diagnosis between NPC and nasopharyngeal lymphoma (NPL) [29]. In terms of the acquisition protocol, the authors demonstrated that a virtual monochromatic reconstruction at 40 keV is optimal in terms of image quality and shows a better demarcation of tumor margins. In addition, it was shown how the combined use of DECT-derived parameters, such as the normalized iodine concentration (NIC), slope of the spectral Hounsfield unit curve, and effective atomic number (Zeff), allowed for better differentiation between NPL and NPC than the evaluation of individual parameters or a morphologic assessment at SECT. Specifically, NPL constantly showed higher DECT parameter values, when compared to NPC. 

Spectral imaging with DECT also appears to be a promising diagnostic tool that can non-invasively allow the preliminary differentiation between malignant and benign tissues [43]. In a retrospective study of 77 patients [48], Wang et al. attempted to define quantitative DECT-derived parameters that could differentiate stage T1 NPC from lymphoid hyperplasia of the nasopharynx (LH), finding that the iodine concentration (IC), normalized iodine concentration (NIC), mean atomic number (Zeff), the attenuation values in the VMIs at 50–190 keV (with 20 keV-interval), particularly at 70 keV, and the slope of the spectral attenuation curve (k) are able to differentiate the two different tissues in a statistically significant manner superior to the analysis with SECT. In the future, this could be a novel approach in the non-invasive differential diagnosis of nasopharyngeal neoplasms.

We also know that the skull base invasion by nasopharyngeal carcinoma is a poor negative prognostic factor for this neoplasm [33,35,43,44], so Zhan et al. analyzed the ability of DECT to more confidently define the neoplastic invasion of skull base bone tissues [36], distinguishing it from alterations attributable to bone sclerosis, showing how there were higher values of the normalized iodine concentration and effective atomic number in sclerosis and lower values in erosion than those in normal bones and how DECT possessed sensitivity, specificity, and diagnostic accuracy, even for early bone involvement, in this implementation that were significantly superior to the simulated SECT and MRI.

Alongside applications with diagnostic and prognostic utility, it appears that spectral imaging may also find its role in defining predictive parameters of the response to therapy. Another study by Zhan et al. conducted on 56 patients sought to evaluate whether parameters derived from DECT could be predictive of an early response to therapy and correlated with survival in patients with NPC [27], showing how NIC and Zeff values can predict an early response to induction chemotherapy and survival in NPC and how a high NIC value correlates with worse survival independently of other factors.

Thus, the use of DECT in the study of nasopharyngeal cancers seems to offer promising results in terms of diagnosis, prognosis, and prediction, especially with the analysis of the parameters that can be derived from the acquisitions, but further investigation is needed before their routine use in the clinic can be considered, especially in relation to cut-off values of the aforementioned DECT-derived parameters. Table 1 presents a short summary of the findings of the studies mentioned in the above paragraph.

## 3. DECT in Cancers of the Oral Cavity

In the group of the head and neck region, carcinoma of the oral cavity is one of the most frequent malignancies and oral squamous cell carcinoma (OSCC) is the most common histotype [49,50,51,52,53]. The main risk factors include smoking and alcohol, but also chronic microtrauma induced by dental anomalies or improperly treated dentures [52,53,54,55]. Many neoplasms arise on precancerous dysplastic lesions such as leukoplakia and erythroplachia, and a small proportion see HPV involved in the pathogenesis [54,55,56,57]. The affected districts may be the lips, palate, floor oral cavity, retromolar trigone, and tongue [54]. The tumor arises as a superficial lesion that tends to ulcerate and invade underlying structures such as muscle and bone, with a marked tendency for lymphatic invasion, through which it reaches the lymph nodes in the neck [54,55,57]. Depending on the stage, clinical condition of the patient, and site, treatment may be surgical and/or radiotherapy possibly associated with chemotherapy [50,51,58]. The proper staging of the patient is, therefore, of paramount importance for the subsequent treatment decision [53]. The imaging evaluation of oral cavity tumors is generally devolved to CT or MRI examinations, but these often pose a considerable challenge for the radiologist, related to the not-always-simple definition of crucial information for the surgeon or oncologist such as the extent and margins of disease, as well as the invasion of closely adjacent structures such as bone and muscle [50,51,52,54,55,57,58,59,60]. This becomes even more relevant in light of the eighth revision of the TNM classification of oral squamous cell carcinomas (OSCC), by far the most frequent malignancy in this district, which includes in the definition of the T parameter, the assessment of depth of invasion (DOI) [53,61]. Moreover, the presence of metal artifacts from dentures often makes image interpretation even more difficult and complicates the correct definition of tumor margins in order to plan precise radiotherapy treatment [62]. Metal artifact reduction (MAR) algorithms applied to DECT acquisitions with VMI reconstructions at 140–200 KeV in evaluating the oral cavity have been shown to be superior to the results achievable with SECT acquisitions [63]. 

In a study by Tanaka et al. [62], different observers evaluated the image clarity and quality of nine patients with oral cancer in terms of metal artifacts due to dental prosthesis, the internal tumor structure, the tumor–organ boundary, and the total quality of images for diagnosis. DECT reconstruction techniques such as iodine density imaging (IDI) and virtual monochromatic imaging (VMI) showed that the estimated tumor volume was not significantly different between VMI and IDI, but, in comparison, the same evaluation on MRI was significantly lowest in three images.

It is now well-known that DECT allows for better quality images to be obtained also for oral cavity tumors, compared with SECT, resulting in a more accurate definition of tumor margins [64]. Recently, the role that DECT can have has emerged, already at a preoperative stage, as a tool for the prognostic stratification of patients based on the evaluation of parameters obtainable from spectral acquisitions: a study conducted on 93 patients by Yang et al. [65], with histologically diagnosed tongue squamous cell carcinoma (OTSCC) at different pathologic stages, histologic differentiation, lymph node statuses, and perineural invasion statuses (PNI), assessed the existence of a correlation between the NIC parameters, slope of the spectral Hounsfield unit curve (λHU), normalized effective atomic number (nZeff), and normalized electron density on both arterial phase (AP) and venous phase (VP) acquisitions. Regarding a correlation with stage of disease, it was shown that λHU and NIC in AP and λHU, nZeff, and NIC in VP were significantly lower in stage III–IV lesions than in stage I–II lesions, while, in relation to histologic grading, it was seen that λHU and NIC were higher in well-differentiated lesions than in poorly differentiated lesions. Regarding the risk of lymph node metastasis, it was seen that λHU and NIC in VP were lower in OTSCCs with lymph node metastasis than those without metastasis. The study, however, failed to find any statistically significant correlation between DECT parameters and the presence of PNI. Table 2 presents a short summary of the findings of the studies mentioned in the above paragraph.

## 4. DECT in Oropharyngeal Cancer

Oropharyngeal carcinoma (OPC) is a significant contributor to the group of head and neck neoplasms. Its epidemiology has been changing, with a rising incidence in developed countries, largely attributed to the Human Papillomavirus (HPV) [58,66,67,68,69]. Squamous cell carcinoma (SCC) is the most common histotype. Oropharyngeal squamous cell carcinoma (OPSCC) is often associated with HPV, leading to a distinct clinical entity compared to non-HPV OPSCC [68,70,71]. It is estimated that HPV forms are 2.5 times more frequent than non-HPV forms [72]. The tumor typically originates in the tonsils or base of the tongue, which is why symptoms such as sore throat or difficulty swallowing are often the first signs of the disease [73]. Risk factors for OPC include HPV infection, smoking, and alcohol consumption [74,75,76]. It is unclear if having HPV alone is enough to cause oropharyngeal cancers, or if other factors (such as smoking or chewing tobacco) interact with HPV to cause these cancers [67,77]. HPV-positive and HPV-negative OPSCC present different risk profiles and clinical outcomes. Prognosis depends on the stage of the tumor, nodal involvement, and histology, with biomarkers such as p16—a surrogate marker for HPV—usually associated with a better prognosis [58,68]. If the cancer is diagnosed at an early stage, the five-year relative survival rate for all people is 86%. If the cancer has spread to surrounding tissues or organs and/or the regional lymph nodes, the five-year relative survival rate is 69% [67]. Treatment primarily involves radiotherapy, often combined with chemotherapy, while surgery is typically reserved for recurrent or persistent disease [67,77,78,79,80,81]. The mortality rate is associated with bleeding complications from both surgery and radiotherapy [82]. Imaging modalities such as MRI are crucial for accurate tumor delineation due to their superior soft tissue contrast but are often supplemented with other imaging techniques such as CT and PET for a comprehensive evaluation [67,69].

As a result of the above, the assessment of HPV status in neoplastic cells plays an extremely important role in the diagnostic and prognostic definition of OPC patients. Currently, it is the histologic examination with an immunohistochemical analysis that defines this parameter, because, although it is known that HPV+ tumors tend to have regular lesion margins and a homogeneous enhancement with lymph node metastases of cystic appearance, unlike HPV- tumors, a morphologic imaging assessment alone is not a sufficient criterion to be able to guide therapeutic decisions [71]. However, the additional information derivable from spectral imaging of DECT could provide new evaluation criteria for establishing the presence of the virus by non-invasive methods. Li et al., in a recent retrospective study of 35 patients [83], showed that the effective atomic number (Zeff), slope of the spectral Hounsfield unit curve calculated on virtual monoenergetic imaging from 40 to 200 keV, and normalized iodine density (NID) of p16+ tumors were all lower than p16- ones, but only NID was statistically able to discriminate the p16- types from positive ones, with a threshold value of 0.495, probably reflecting differences in the microvascular structure of the two different tumor forms. The study, although not conducted on tumors located exclusively in the oropharynx, makes clear the possibility that DECT can realistically help in defining such an important parameter for prognostic purposes. Table 3 presents a short summary of the findings of the studies mentioned in the above paragraph.

## 5. DECT in Laryngeal Tumors

Laryngeal squamous cell carcinoma (LSCC) is the most common tumor of the upper aerodigestive tract [84,85]. The most common histotype is squamous cell carcinoma, which recognizes tobacco exposure and alcohol as the main risk factors, as well as precancerous lesions such as vocal cord leukoplakia or papillomatosis [86,87]. The larynx is divided into supraglottic, glottic, and subglottic regions, and the location of the lesion in one of these areas has different prognoses, mainly due to the different distribution of the lymphatic network and the different timing of symptom onset [86,88,89]. In relation to the crucial role that the organ plays in the relational life of patients, over time, organ-preserving treatments have become increasingly established, either through a combined RT-CHT approach or through conservative surgery (partial laryngectomy or with the use of laser), depending on the stage of disease at diagnosis, relegating total laryngectomy to cases of therapeutic failure (salvage surgery) or advanced disease cases [84,85,86,87,90].

An accurate diagnosis, both through endoscopic examination and imaging, are fundamental requirements for the correct staging of the patient and, therefore, for the best therapeutic indication [91,92,93,94,95]. The imaging examination by CT is generally the most indicated for the evaluation of tumors in this site as it, compared to MRI, is less affected by respiratory motion artifacts due to the different acquisition time. However, MRI has a greater diagnostic sensitivity in evaluating the infiltration of the paraglottic space due to its better contrast resolution and is considered superior to CT for evaluating the infiltration of the thyroid cartilage [92,93,94,96]. The eighth revision of the TNM classification of laryngeal tumors highlights the importance of evaluating the infiltration of the thyroid cartilage as it can determine stage T3 or the transition to stage T4a [97]. The ability to accurately assess the actual extent of the primary tumor on imaging, as well as being able to accurately determine the degree of invasion of the thyroid cartilage are essential elements for a correct prognostic stratification of the patient. DECT applied to the study of laryngeal tumors offers numerous advantages when compared to what can be obtained with conventional SECT acquisitions in the locoregional evaluation of the disease. Firstly, it improves the detection of the lesion and allows a more precise evaluation of its relative loco-regional extension for staging purposes as a result of the use of virtual monochromatic (VMI) reconstructions capable of highlighting, in relation to the surrounding structures, the tumor tissue; this has been seen to be true for iodine overlay maps and, both in terms of image quality and diagnostic accuracy, for VMI reconstructions at 40–50 KeV [98,99,100]. Secondly, it has been seen to be able to more accurately differentiate an early glottic squamous cell carcinoma from a precancerous lesion such as leukoplakia or simple chronic inflammation [101]. Finally, it significantly improves, compared to SECT, the accuracy in evaluating the cartilage invasion by the tumor, combining the advantages of CT (less sensitivity to motion artifacts) with those of MRI. In this regard, Kuno et al., in a study with 55 patients, showed that DECT has a higher specificity and acceptable sensitivity in diagnosing the laryngeal cartilage invasion compared with MR imaging [92].

The advantages of spectral analysis possible as a result of DECT acquisitions, however, seem to be able to go even further, opening the doors to a more accurate non-invasive prognostic stratification of patients with LSCC. In particular, Wang et al. evaluated whether pre-operative DECT could provide predictive elements on the expression of Ki-67 by the tumor, a biomarker related to prognosis and survival, showing, in a retrospective study on 88 patients, that the DECT-derived parameters IC, NIC, Zeff, attenuation values in VMIs 40–80 keV, and slope of the spectral Hounsfield unit curve were positively correlated with the Ki-67 expression, with the NIC value having the highest correlation among the others, and that all parameters were significantly higher in LSCC with a high Ki-67 expression than in those with a low Ki-67 expression, making them potentially usable as predictors of survival and prognosis in LSCC [102].

Similar conclusions were reached in a work by Geng et al. [84] in which 65 patients were retrospectively evaluated in search of a correlation between histopathological prognostic factors such as tumor grading, T stages, and N stages, and DECT-derived parameters, demonstrating the existence of a statistically significant correlation between high values of the iodine concentration (IC) and normalized IC (NIC) of the tumor calculated in the arterial phase with moderately and poorly differentiated lesions, with higher T stages and positivity compared to lymph node metastasis [84]. Shen et al., in another retrospective study on 72 patients with HNSCC, showed that the parameters NIC, λHU, NZeff, and the attenuation value on the noise-optimized virtual monoenergetic image (VMI+) at 40 keV were predictive for prognostic factors such as tumor grading, lymphovascular invasion, and perineural invasion [103].

Patients with LSCC who are candidates for conservative surgical treatment are burdened by a high risk of locoregional recurrence variable in the literature from 30 to 66% [104], lower in patients with an early-stage neoplasm [89]. Bahig et al. had already investigated the possibility, on a small sample of patients, of being able to use DECT parameters as predictive factors of the risk of recurrence after conservative surgery and adjuvant radiotherapy in patients with LSCC, highlighting an increase in risk at high values of iodine concentration (IC) at pre-treatment DECT [25]. In a subsequent study by Zhang et al. [6] on a larger sample of patients, it was investigated whether, in early-stage glottic tumors, with an indication for conservative treatment, it was possible to develop a model comprising DECT-derived parameters that would allow us, in the pre-treatment stage, to predict the risk of post-operative recurrence (RFS—recurrence-free survival). The retrospective analysis of 212 patients showed that high values of NIC calculated in arterial and venous phases were significant predictors of RFS [6]. Although, among head and neck tumors, the larynx is one of the areas in which DECT has been most studied, further studies are necessary before these results can be applied clinically. Please refer to Table 4 for a summary of the findings cited in the above paragraph.

## 6. DECT in the Evaluation of Neck Lymph Nodes Metastasis

Lymph nodes are the most common site of metastasis from head and neck neoplasms [65,66,105]. It is not uncommon for lymph node metastasis to be the first manifestation of the presence of a neoplasm in this district, beginning clinically as a laterocervical swelling, the primary site of which sometimes cannot be identified either by pan-endoscopy or imaging [65,106,107,108]. Because of the more frequent neoplastic histotype in head–neck districts, lymph node metastases are usually squamous cell carcinomas [108]. The presence of lymph node metastasis, the number of lymph nodes involved, and extra-capsular extension are the main factors affecting patients’ prognosis and survival [106,107,109]. From this, we can deduce the importance of imaging in the evaluation of lymph node sites and the need for increasingly advanced techniques that can improve diagnostic accuracy with the aim of reducing the use of invasive diagnostics in order to be able to identify the most appropriate treatment. Generally, the study of lymph nodes is conducted in preoperative CT where they are evaluated based on morphological and structural features such as size, margins, density, extracapsular extension, and enhancement [109]. Especially when lymph nodes are small in size, however, we know that this analysis is almost never sufficient to be able to make a diagnosis, and, sometimes, lymph nodes that do not show suspicious features are actually metastatic [108]; therefore, the combined evaluation through various other imaging methods (MRI, PET, US, and CT) is the most adopted approach [109]. Some authors proposed scores that, by combining different morphological parameters, had a predictive value on the risk of the presence of lymph node metastasis [110]. However, because of these limitations and non-standardized scores, patients often undergo lymph node dissections of the neck as a precautionary measure, resulting in invasive treatments to patients who may not benefit [49,50,105]. DECT, with the potential offered by spectral imaging, enabling enhanced material differentiation and quantification, could allow the current interpretative limitations of SECT images to be overcome. The extensive application of DECT in the study of tumors has also highlighted its potential in the evaluation of lymph node metastases [111,112]. Not many studies have investigated its role in the context of squamous cell neoplasms of the head and neck district, probably given the relative rarity of these neoplasms and the relatively small distribution of DECT scanners, so the information we have is largely extrapolated from studies conducted on lymph node metastases of other cancers [112]. It is enough to mention that the first study in the literature on the extensive use of DECT-derived parameters in the evaluation of lymph nodes in patients with oral cavity tumors was in 2018 by Foust et al. [26], who, albeit on a small group of patients, had demonstrated the existence of a correlation between spectral parameters and metastatic lymph node positivity. Previous studies conducted in that district were largely limited to assessments of different iodine concentrations in metastatic versus inflammatory or normal lymph nodes quantifiable by DECT scans [109]. Luo et al. [51] has, in this, sought to confirm the potential of DECT, already observed in other districts, also in the case of lymph node metastasis from oral cavity cancer, demonstrating, in a study of 103 patients undergoing surgical treatment of the primary tumor and lymph node resection of levels I–III, for a total of 399 lymph nodes, that the values of ED, IC, NIC, λHU, and the dual-energy index (DEI) were significantly different between the metastatic and non-metastatic lymph nodes, and, in particular, the association between NIC values and morphologic values such as diameter had a higher diagnostic accuracy than any other parameter taken individually (Table 5). In contrast, no difference was observed between positive and negative lymph nodes based on Zeff values.

## 7. DECT in Head and Neck Squamous Cell Carcinoma: New Frontiers

Radiomics is an emerging field that involves the extraction of quantitative features from medical images using data-characterization algorithms. These radiomics features can capture information about the shape, texture, and intensity of tumors that may not be visible to the naked eye. The goal of radiomics is to uncover patterns and correlations between features that may be indicative of disease characteristics and outcomes [113]. In the context of head and neck tumors, radiomics has shown great promise in improving the accuracy of diagnosis, prognosis, and treatment planning. By analyzing imaging data from DECT, radiomics can provide insights into tumor heterogeneity, which is a key factor in understanding tumor behavior and response to therapy. The integration of radiomics features with clinical and genomic data can lead to the development of predictive models that support personalized medicine [112,114].

In recent years, not many studies in literature have further explored the role of radiomics in combination with DECT imaging in head and neck cancers. Li et al. developed the DECT-based radiomics nomogram [115] using radiomics-signature models built upon the feature extracted on VMI and iodine-based material decomposition images (IMDI) that, combined with clinical data, showed the ability to predict poorly differentiated from moderately well-differentiated HNSCC. In another study, Bernatz et al. [116] evaluated whether, through spectral data obtained by DECT material decomposition, it was possible to improve radiomics-based predictive models of survival in HNSCC, finding, in a preliminary evaluation, that there was no significant increase in the accuracy of these models. Zhang et al. [117] have tried to combine the advantages of radiomics with a DECT spectral analysis in the study of the lymph node metastasis of head and neck cancers in order to develop nomograms predictive of the risk of lymph node positivity, obtaining clinically meaningful results. This makes it clear that, alongside new techniques for acquiring diagnostic images and new quantitative methods for analyzing them, there is a potential benefit in their combined application, although, for each, the actual significance should be carefully assessed.

In addition to radiomics, clustering techniques and machine learning play a crucial role in the analysis of imaging data and help in reducing the dimensionality of the data and enhancing the interpretability of the results [118]. Clustering techniques, such as k-means and hierarchical clustering, are used to group similar radiomics features, facilitating the identification of distinct tumor phenotypes [119]. Chamroukhi et al. [120] propose novel unsupervised learning techniques using functional data analysis and mixture models to cluster DECT images. These methods integrate spatial image context and spectral information from DECT scans to improve tumor segmentation and characterization. To our knowledge, this is the only article evaluating the application of clustering techniques on DECT imaging in the context of head and neck cancers. The study evaluates the proposed methodology on 91 DECT scans of HNSCC tumors, comparing the results to manually traced tumor contours and other baseline algorithms. The findings suggest that the proposed clustering methods can enhance the accuracy and consistency of tumor delineation and characterization, potentially aiding in clinical outcome prediction and improving the overall evaluation of head and neck cancers.

## 8. Discussion

In recent years, DECT has emerged as a transformative imaging modality in the evaluation of HNSCC. The introduction of DECT has provided clinicians and researchers with a powerful tool for the non-invasive assessment of head and neck cancers. By offering material-specific imaging and quantitative analysis, DECT facilitates a more nuanced approach to diagnosis and treatment planning. This is particularly relevant in the context of HNSCC, where the accurate staging and characterization of tumors are crucial for optimal patient management.

The current body of research on the application of DECT in the evaluation of head and neck squamous cell carcinomas (HNSCCs) is promising yet nascent. Despite this promise, the literature on its application in HNSCC remains limited. The number of studies to date, often limited by their retrospective nature and small sample sizes, reflects the relative rarity of malignancies of the head and neck region and the limited diffusion of DECT scanners among centres. Despite these limitations, the findings suggest a significant potential for DECT in improving the diagnostic accuracy, lesion characterization, and treatment monitoring for these cancers, potentially leading to better patient outcomes. DECT represents a significant advancement in CT technology, offering enhanced diagnostic capabilities in oncologic imaging. These techniques have shown promise in improving image quality, increasing lesion detectability, and reducing dental artifacts, which are particularly beneficial in the complex anatomy of the head and neck region. Furthermore, the possibility of being able to derive quantitative parameters from spectral analysis will increasingly allow a multiparametric evaluation of pathological tissue alterations, similar to what happens with MRI. Combining these advantages with the new frontiers made possible by radiomics demonstrated great potential in preoperative predictions, aiding in the development of individualized treatment plans. The ability to quantify the degree of lesion enhancement through iodine mapping could revolutionize the assessment of tumor infiltration and lymph node status, which are critical factors in treatment planning and prognosis. The integration of novel techniques such as radiomics, clustering, and machine learning in the analysis of head and neck tumors represents a significant advancement in the field of medical imaging. These approaches offer a deeper understanding of tumor biology and provide valuable tools for personalized medicine. As research in this area continues to evolve, the combination of DECT and machine learning holds great promise for improving patient care and outcomes.

Table 6 offers an overall summary of all the results of the studies evaluated in this review.

## 9. Conclusions

As the field of DECT in HNSCC continues to evolve, it is evident that more extensive research is required to validate these initial findings and to establish standardized protocols for clinical practice. The integration of DECT into routine diagnostic workflows could significantly impact patient outcomes by providing more precise and personalized care for those affected by head and neck squamous cell carcinomas.

## Figures and Tables

**Table 1:** Summary of the findings of papers studying DECT application in nasopharyngeal cancer.

Authors	Aim of the Study	DECT-Parameters	Findings
Shen et al. [29]	Use of DECT to differentiate NPC from NPL.	NIC λHU Zeff	Combined parameters can differentiate NPC from NPL. VMI+ at 40 KeV was optimal in detection of tumors.
Wang et al. [48]	Role of DECT in differentiating stage T1 NPC from LH of the nasopharynx.	IC NIC Zeff λHU HU in VMI	All parameters had higher value in T1 NPC. Combining those parameters results in high diagnostic accuracy.
Zhan et al. [36]	Role of DECT in defining neoplastic invasion of skull base bone tissues, comparing to simulated SECT and MRI.	NIC Zeff	DECT parameters have higher value in bone sclerosis and lower in lytic lesions. DECT is better than simulated SECT and MRI in detecting skull base invasion.
Zhan et al. [27]	Role of DECT in predicting response to therapy and survival.	NIC Zeff	Parameters predict response to therapy and survival; high NIC value is an independent predictive factor of poor survival.

NPC = nasopharyngeal carcinoma; NPL = nasopharyngeal lymphoma; LH = lymphoid hyperplasia; NIC = normalized iodine concentration; λHU = slope of the spectral Hounsfield unit curve; VMI = virtual monochromatic image; Zeff = effective atomic number; IC = iodine concentration; HU = Hounsfield unit.

**Table 2:** Summary of the findings of papers studying DECT application in cancer of the oral cavity.

Author	Aim	DECT-Parameters	Findings
Tanaka et al. [62]	Comparing DECT VMI, iodine density map, and MRI.	/	DECT imaging were better in estimating tumor volume than MRI. Iodine density image quality is superior to VMI.
Laukamp et al. [63]	Comparing metal artifact reduction algorithms/reconstructions from spectral detector to conventional imaging.	/	Metal artifact reduction algorithms and reconstructions granted significant artifact reduction compared to conventional CT images.
Toepker et al. [64]	Comparing DECT imaging to single-energy images at 80 kV and 140 kV in oral tumors.	/	DECT showed higher image quality and higher SNR compared to SECT imaging.
Yang et al. [65]	Role of DECT as prognostic tool in patient with OTSCC.	NIC λHU nZeff nED	DECT-derived parameters calculated both AP and VP, and showed significant correlation with pathologic stages, histologic differentiation, lymph node status, and perineural invasion.

VMI: virtual monochromatic reconstructions; SNR: signal-to-noise ratio; OTSCC: oral tongue squamous cell carcinoma.

**Table 3:** Summary of the findings of papers studying DECT application in cancer of the oropharynx.

Author	Aim	DECT-Parameters	Findings
Li et al. [83]	Evaluate differences in DECT between p16(+) and p16(−) HNSCC.	Zeff λHU NIC	All parameter values in p16(+) tumors were significantly lower than p16(−) ones. NIC alone were able to discriminate p16(−) from p16(+) HNSCC (AUC = 0.788) with a threshold value of 0.495.

HNSCC: head and neck squamous cell carcinoma; NIC: normalized iodine concentration; λHU: slope of the spectral Hounsfield unit curve; Zeff: effective atomic number; AUC: area under the curve.

**Table 4:** Summary of the findings of papers studying DECT application in laryngeal cancer.

Author	Aim	DECT-Parameters	Findings
Zheng et al. [98]	Finding best VMI to detect LHSCC and assess diagnostic performance.	/	Image quality of VMIs 40–50 keV is higher than conventional CT imaging; VMI 40 keV has better diagnostic accuracy than conventional CT imaging.
Zopfs et al. [99]	Evaluate diagnostic value of iodine overlay maps and VMI for initial assessment of HNSCC.	/	Iodine overlay maps and low-energy VMI improve initial assessment of tumor compared to conventional images.
He at al. [100]	Assess image quality of laryngeal SCC using DECT reconstruction algorithms.	/	VMI+ 40 keV and NBI improve image quality of laryngeal SCC.
Wang et al. [101]	Evaluate role of DECT in discriminating eGSCC from chronic inflammation and leucoplakia of the vocal cord; comparing diagnostic efficiency of DECT with simulated conventional CT.	IC NIC Zeff HU in VMIs 40–100 keV λHU	Parameters showed higher value in eGSCC. NIC and attenuation at 60 keV are more able to discriminate glottic lesion than simulated SECT.
Kuno et al. [92]	Comparing diagnostic accuracy of MRI and DECT images in detecting cartilage invasion by laryngeal and hypopharyngeal squamous cell carcinomas.	/	DECT showed higher specificity than MRI for diagnosing cartilage invasion, and sensitivity does not differ significantly.
Wang et al. [102]	Predicting Ki-67 expression by dual-energy CT in laryngeal squamous cell carcinoma.	IC NIC Zeff HU value in VMIs 40–80 keV λHU	Parameters positively correlated with Ki-67 expression; values were significantly higher in high Ki-67 tumors than in low Ki-67 ones.
Geng et al. [84]	Correlation between DECT-parameters and tumor grading, and T and N stages.	IC NIC	Both parameters calculated in arterial phase were higher in poorly differentiated tumors, higher T stages, and N stages.
Shen et al. [103]	Predicting histopathological features with DECT parameters.	NIC λHU nZeff A_40_	Parameters showed higher value in high-grade tumors, and those with lymphovascular and perineural invasion

HNSCC = head and neck squamous cell carcinoma; LHSCC = laryngeal and hypopharyngeal squamous cell carcinoma; eGSCC = early glottic squamous cell carcinoma; NIC = normalized iodine concentraton; IC = iodine concentration; λHU = slope of the spectral Hounsfield unite curve; VMI = virtual monochromatic image; Zeff = effective atomic number; nZeff = normalized effective atomic numbers; HU = Hounsfield unit; NBI = nonlinear blending image; A40 = attenuation value in VMI 40 KeV.

**Table 5:** Summary of the findings of papers studying DECT application in head and neck lymph node evaluation.

Author	Aim	DECT-Parameters	Findings
Foust et al. [26]	Role of DECT parameters as predictors of nodal metastasis in OPSCC.	IC λHU	Both parameters were lower in metastatic lymph nodes.
Tawfik et al. [109]	Evaluate difference in DECT parameters between normal, inflammatory, and metastatic squamous cell carcinoma cervical lymph nodes.	IC IO	DECT-derived IC and IO differ significantly among normal, inflammatory, and metastatic SCC cervical lymph nodes.
Luo et al. [51]	Potential of DECT parameters in identifying metastatic cervical lymph nodes in oral squamous cell carcinoma.	ED IC NIC λHU DEI	All parameters showed significantly decreased values in metastatic nodes.

HNSCC = head and neck squamous cell carcinoma; OPSCC = oropharyngeal squamous cell carcinoma; NIC = normalized iodine concentration; IC = iodine concentration; λHU = slope of the spectral Hounsfield unite curve; Zeff = effective atomic number; ED = electron density; HU = Hounsfield unit; IO = iodine overlay; DEI = dual-energy index; AP = arterial phase; VP = venous phase; LNM = lymph node metastasis.

**Table 6:** Summary of all the papers analyzed in this review.

Authors	Aim of the Study	Findings
Shen et al. [29]	Use of DECT to differentiate NPC from NPL.	Combined parameters can differentiate NPC from NPL.
Wang et al. [48]	Role of DECT in differentiating stage T1 NPC from LH of the nasopharynx.	Combined parameters can differentiate T1 NPC from LH.
Zhan et al. [36]	Role of DECT in defining bone invasion vs. SECT and MRI.	DECT parameters can differentiate lytic lesion from sclerosis and it is better than conventional imaging in detecting skull base invasion.
Zhan et al. [27]	Role of DECT in predicting response to therapy and survival.	Parameters predict response to therapy and survival.
Tanaka et al. [62]	Comparing DECT VMI, iodine density map and MRI.	DECT imaging were better in estimating tumor volume than MRI.
Laukamp et al. [63]	Comparing MAR algorithms/reconstructions from spectral detector to conventional imaging.	MAR algorithms and reconstructions granted significant best artifact reduction.
Toepker et al. [64]	Comparing DECT imaging to SECT images at 80 kV and 140 kV in oral tumors.	DECT showed higher image quality and higher SNR compared to SECT.
Yang et al. [65]	Role of DECT as prognostic tool in patient with OTSCC.	DECT parameters showed correlation with pathologic stages, histology, N status, and PNI.
Li et al. [83]	Evaluate differences in DECT between p16(+) and p16(−) HNSCC.	DECT parameter values in p16(+) tumors were significantly lower than p16(−) ones.
Zheng et al. [98]	Finding best VMI to detect LHSCC and assess diagnostic performance	VMI 40 keV has better diagnostic accuracy than conventional CT imaging.
Zopfs et al. [99]	Evaluate diagnostic value of iodine overlay maps and VMI for initial assessment of HNSCC.	Iodine overlay maps and low-energy VMI improve initial assessment of tumor.
He at al. [100]	Assess image quality of laryngeal SCC using DECT reconstruction algorithms.	VMI+ 40 keV and NBI improve image quality of laryngeal SCC.
Wang et al. [101]	Evaluate role of DECT to discriminate eGSCC from chronic inflammation and leucoplakia of the vocal cord.	DECT parameters showed higher value in eGSCC, discriminating glottic lesion, than simulated SECT.
Kuno et al. [92]	Comparing MRI and DECT in detecting cartilage invasion by LHSCC.	DECT showed higher specificity than MRI for diagnosing cartilage invasion.
Wang et al. [102]	Predicting Ki-67 expression by DECT in LSCC.	DECT parameters positively correlated with Ki-67 expression.
Geng et al. [84]	Correlation between DECT-parameters and tumor grading, and T and N stages.	DECT parameters were higher in poorly differentiated tumors, higher T stages, and N stages.
Shen et al. [103]	Predicting histopathological features with DECT parameters.	DECT parameters showed higher value in high-grade tumors, LVI, and PNI.
Foust et al. [26]	Role of DECT parameters as predictors of nodal metastasis in OPSCC.	DECT parameters were lower in metastatic lymph nodes.
Tawfik et al. [109]	Evaluate difference in DECT parameters between normal, inflammatory, and metastatic squamous cell carcinoma cervical lymph nodes.	DECT parameters differ significantly among normal, inflammatory, and metastatic SCC cervical lymph nodes.
Luo et al. [51]	Potential of DECT parameters in identifying metastatic cervical lymph nodes in OSCC.	All parameters showed significantly decreased values in metastatic nodes.
Li et al. [83]	Evaluating DECT-based radiomics nomogram to assess tumor differentiation.	DECT-based radiomics nomogram predict poor differentiated tumor from well-differentiated ones.
Bernatz et al. [116]	Evaluate whether DECT material decomposition improves predictive models of survival based on radiomics.	Adding material decomposition data did not increase accuracy of radiomics model.
Chamroukhi et al. [120]	Propose a method based on clustering techniques on DECT images in HNSCC.	Clustering methods can enhance the accuracy and consistency of diagnosis.
Zhang et al. [117]	Validate radiomics to predict lymph node metastasis in HNSCC.	Radiomics models were predictive of LNM.

NPC = nasopharyngeal carcinoma; NPL = nasopharyngeal lymphoma; LH = lymphoid hyperplasia; VMI = virtual monochromatic image; MAR = metal artifact reduction; SNR: signal-to-noise ratio; OTSCC: oral tongue squamous cell carcinoma; PNI = perineural invasion; LVI = lymphovascular invasion; HNSCC = head and neck squamous cell carcinoma; LHSCC = laryngeal and hypopharyngeal squamous cell carcinoma; eGSCC = early glottic squamous cell carcinoma; NBI = nonlinear blending image; LSCC = laryngeal squamous cell carcinoma; OPSCC = oropharyngeal squamous cell carcinoma; OSCC = oral squamous cell carcinoma; LNM = lymph node metastasis.
